# Supplementary figures and images for: Arsenic toxicity in the Drosophila brain at single cell resolution
Source: Front Toxicol. 2025 Jul 10;7:1636431. doi: 10.3389/ftox.2025.1636431 (PMC12287011; doi:10.3389/ftox.2025.1636431)

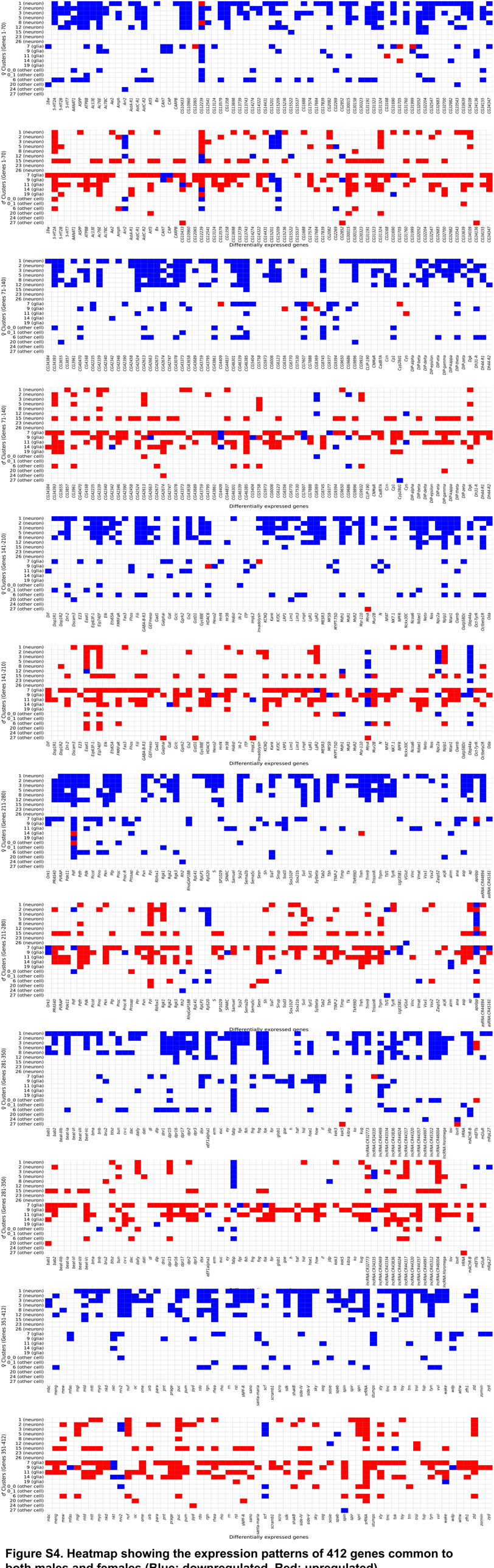

Supplement: Supplementary file 3 [file DataSheet4.pdf]
