# Supplementary material for: Arsenic toxicity in the Drosophila brain at single cell resolution
Source: Front Toxicol. 2025 Jul 10;7:1636431. doi: 10.3389/ftox.2025.1636431 (PMC12287011; doi:10.3389/ftox.2025.1636431)
Supplement: Supplementary file 6 [file DataSheet3.pdf]

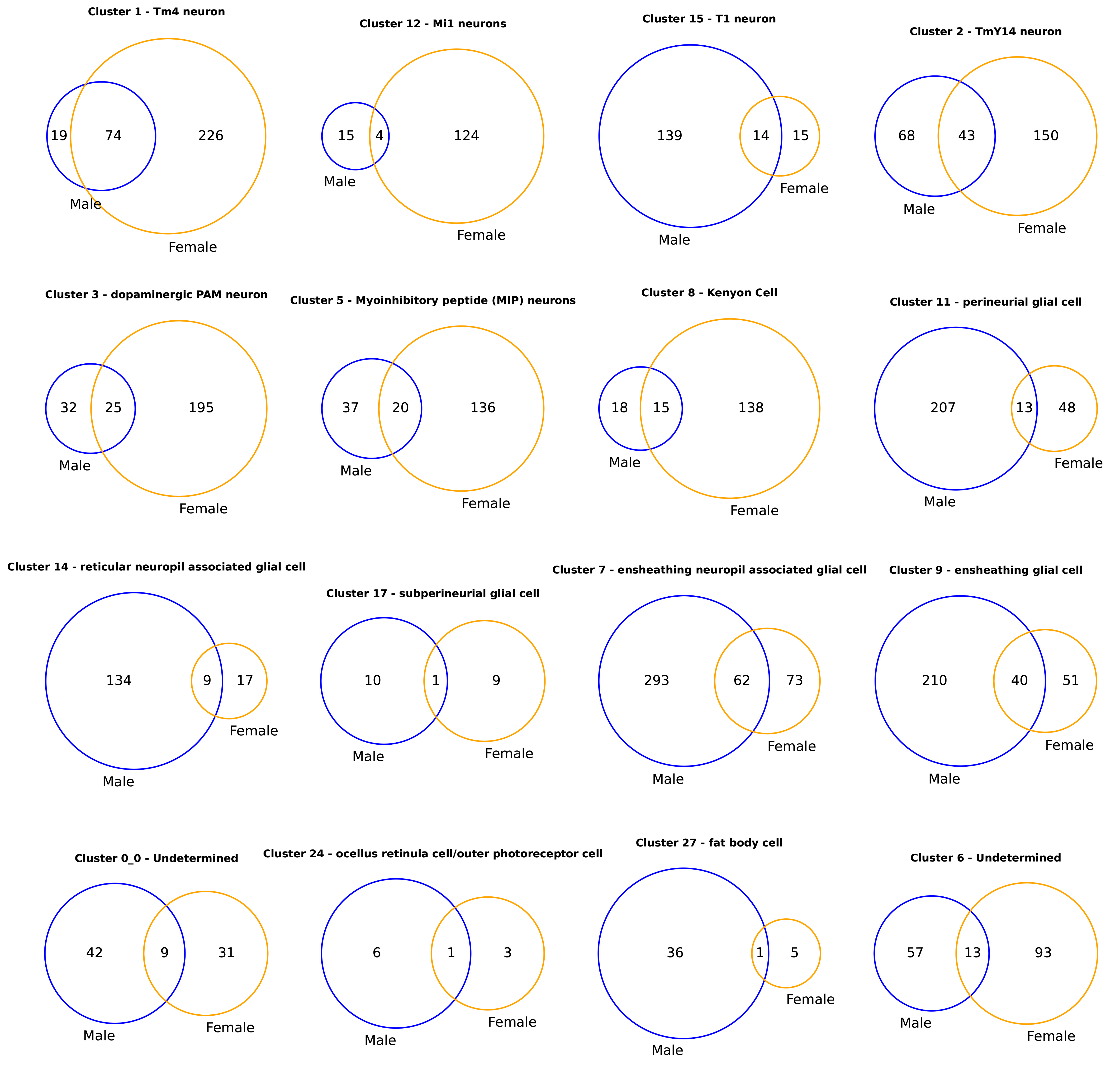

Figure S3. Venn diagram that illustrates the differentially expressed genes (DEGs) that are shared and unique between males and females for each cluster separately.
