# Supplementary material for: Arsenic toxicity in the Drosophila brain at single cell resolution
Source: Front Toxicol. 2025 Jul 10;7:1636431. doi: 10.3389/ftox.2025.1636431 (PMC12287011; doi:10.3389/ftox.2025.1636431)
Supplement: Supplementary file 8 [file DataSheet1.pdf]

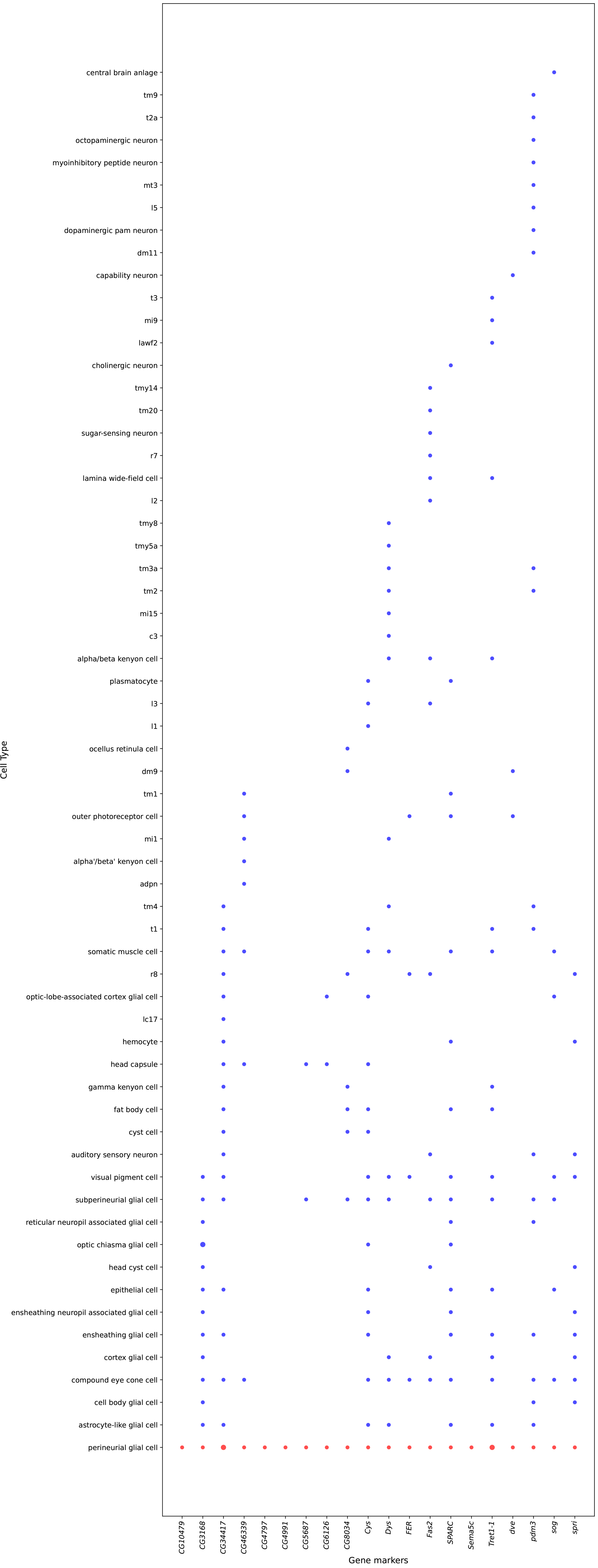

Figure S1. Example of cell cluster annotation. Cluster 11 was annotated as perineurial glial cells based on the BGEE frequency of gene expression for the top 20 marker genes, all of which were exclusively expressed in a single cell type.
