# Supplementary material for: Arsenic toxicity in the Drosophila brain at single cell resolution
Source: Front Toxicol. 2025 Jul 10;7:1636431. doi: 10.3389/ftox.2025.1636431 (PMC12287011; doi:10.3389/ftox.2025.1636431)
Supplement: Supplementary file 9 [file DataSheet5.pdf]

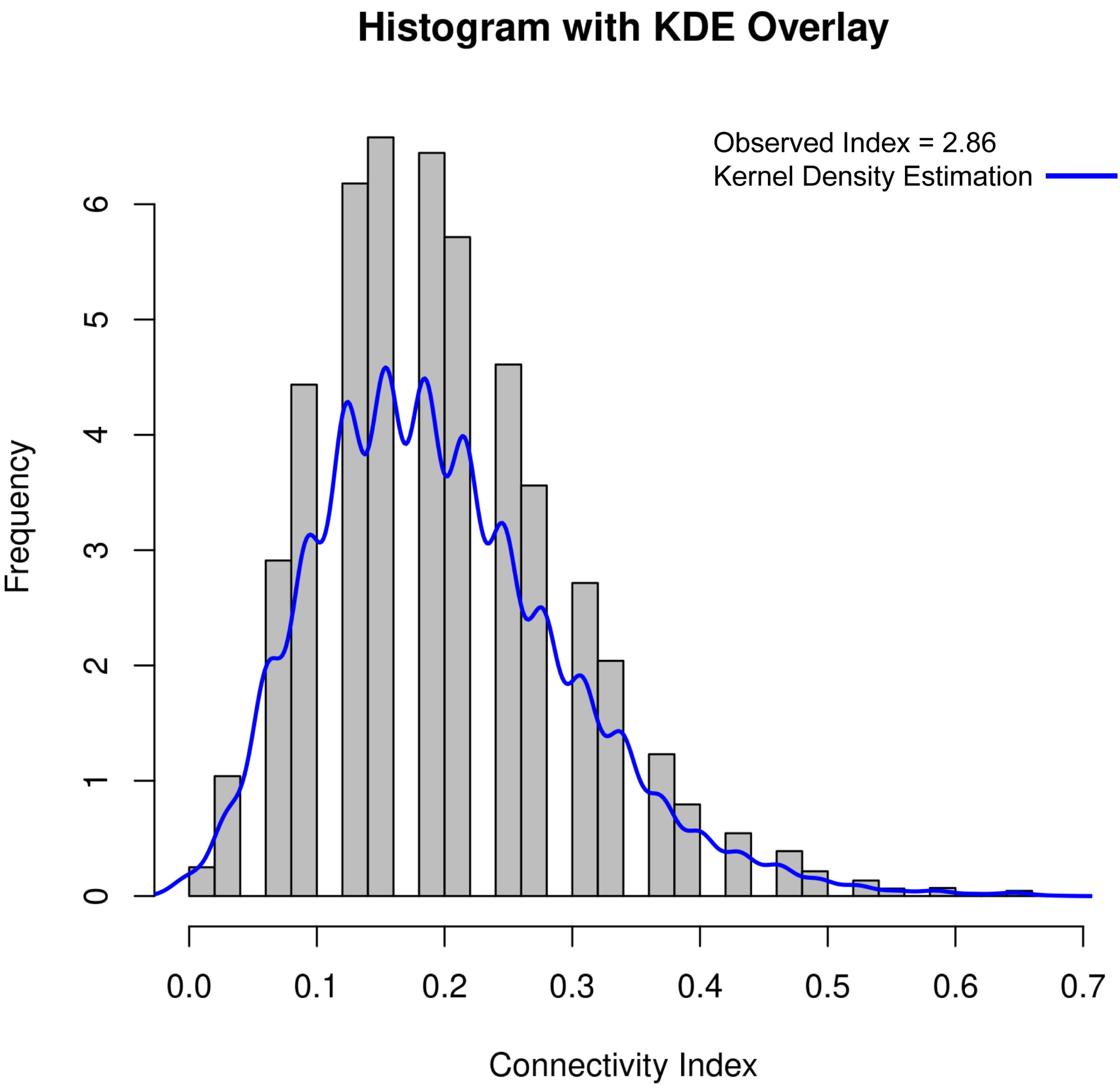

Figure S5: Histogram of the null distribution with kernel density estimation overlay calculated for network connectivity indices from the randomly sampled subnetworks.
